# The complete mitochondrial genome of the hybrid offspring *Epinephelus awoara♀* × *Epinephelus tukula♂*

**DOI:** 10.1080/23802359.2020.1721356

**Published:** 2020-02-03

**Authors:** Ziqi Li, Yongsheng Tian, Zhentong Li, Shuai Chen, Linna Wang, Linlin Li, Jingjing Zhang, Yuping Wu, Zunfang Pang, Wenhui Ma, Jieming Zhai

**Affiliations:** a Key Laboratory of Sustainable Development of Marine Fisheries, Ministry of Agriculture and Rural Affairs, Yellow Sea Fisheries Research Institute, Chinese Academy of Fishery Sciences, Qingdao, China;; b Laboratory for Marine Fisheries Science and Food Production Processes, Qingdao National Laboratory for Marine Science and Technology, Qingdao, China;; c College of Fisheries and Life Science, Shanghai Ocean University, Shanghai, China;; d Chinese Academy of Agricultural Sciences, Beijing, China;; e College of Fisheries and Life Science, Dalian Ocean University, Dalian, China;; f Laizhou Mingbo Aquatic Co., Ltd, Yantai, China

**Keywords:** Mitochondrial genome, *Epinephelus awoara*♀ × *Epinephelus tukula*♂

## Abstract

The complete mitochondrial genome of hybrid grouper from *Epinephelus awoara* (♀) ×*E. tukula* (♂) was obtained by PCR amplification. The circular genome was 16,801 bp in length, consisting of 13 protein-coding genes, 22 transfer RNA genes, 2 ribosomal RNA genes, and a control region (D-loop region). The overall base composition was as follows: A: 28.46%, T: 27.27%, C: 27.27%, G: 16.49%. The new results may provide valuable data for the genetic and taxonomic research on artificial hybrid grouper.

*Epinephelus awoara* and *E. tukula* are both to the subfamily Epinephelinae, family Serranidae and orders Perciformes. *E. awoara* lives in rocky areas as well as on sandy-mud bottoms, mainly distributed in Northwest Pacific: Korea, Japan, China and the like. It is not only a marine fish of high economic value but also an ornamental fish in some countries because of its gorgeous body color. *E. tukula* is mainly found in deep reef channels and seamounts, mainly distributed in Indo-West Pacific: Red Sea and East Africa to southern Japan, Australia, Paracel Islands in the South China Sea, with strong disease resistance and fast growth rate. For the past few years, hybridization was commonly used in fish breeding as it allowed for a combination of advantageous traits from different species (Cheng et al. 2019). And the newly hybrid grouper was obtained by artificial insemination from *E. awoara* (♀) × *E. tukula* (♂). This hybrid offspring has the advantages of low deformity rate and fast growth rate, and has potential economic value, but its genetic characteristics remain poorly understood (Li et al. 2019). Therefore, the complete mitochondrial genome of hybrid of *E. awoara* (♀) ×*E. tukula* (♂) was sequenced to provide useful information for the genetic and taxonomic research on artificial hybrid grouper.

In this study, the sample was collected at Laizhou Mingbo Aquatic Co., Ltd., Shandong province, China (372506.7300 N 1200015.1100E). The sample was numbered QJ2 and stored in a −80 °C refrigerator with accession number 20190705QJ before sequencing. Total DNA was extracted with standard phenol-chloroform methods (Sambrook et al. 1989). The complete mitochondrial genome of this sample (GenBank, MN879323) is 16,801bp in length which was sequenced by high-throughput sequencing technology (illumine HiSeq 2000, USA). It consisted of 13 protein-coding genes, 22 transfer RNA genes, 2 ribosomal RNA genes, and a control region (D-loop region). Overall base composition of the complete mitochondrial DNA is A: 28.46%, T: 27.27%, C: 27.27%, G: 16.49%.

The phylogenetic tree was reconstructed based on the complete mitochondrial genome nucleotide sequences of the hybrid of *E. awoara*♀ × *E. tukula*♂ and 3 families, 12 genera, 29 species using the neighbor-joining (NJ) methods in MEGA 7. The mitochondrial genome sequences were aligned using Seqman and subsequently edited and trimmed. Numbers on each node are bootstrap values of 1000 replicates. As shown in Figure 1, the hybrid of *E. awoara*♀ × *E. tukula*♂ had a closer relationship with *E. awoara* (female parent) than the other species, which demonstrated that the mitochondrial genome DNA of the hybrid was also maternal inherited. In addition, the hybrid had close genetic distance with Carangoides equula of *Carangidae* family, while they were belong to deferent families. This results deserved further study.

**Figure 1.**
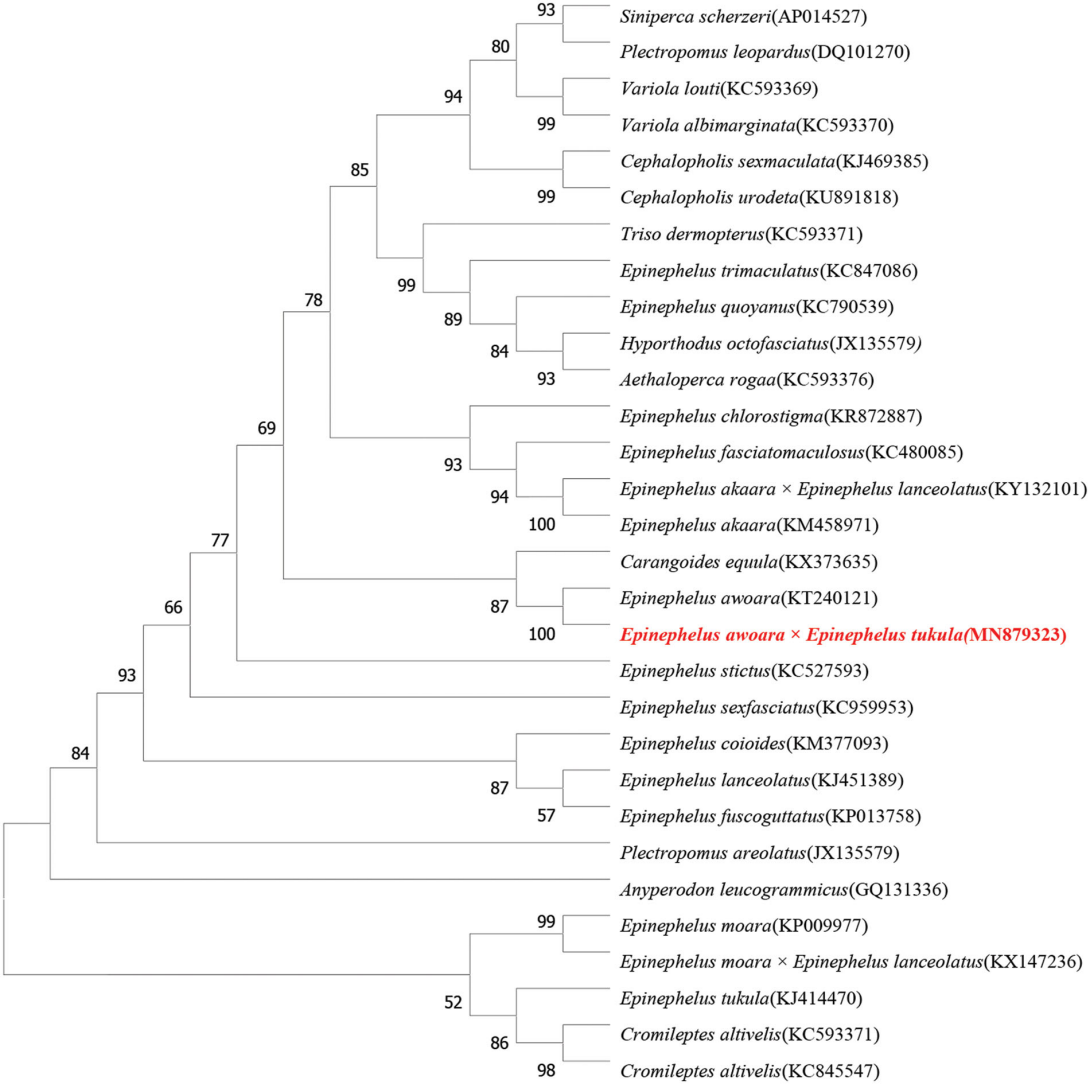
Phylogenetic tree based on mitochondrial genome nucleotide sequences of the hybrid of *Epinephelus awoara*♀ × *Epinephelus tukula*♂ and other 29 species using the NJ methods. Numbers on each node are bootstrap values of 1000 replicates.
